# Fluorescence Spectroscopy Applied to Monitoring Biodiesel Degradation: Correlation with Acid Value and UV Absorption Analyses

**DOI:** 10.1155/2018/4175843

**Published:** 2018-02-27

**Authors:** Maydla dos Santos Vasconcelos, Wilson Espíndola Passos, Caroline Honaiser Lescanos, Ivan Pires de Oliveira, Magno Aparecido Gonçalves Trindade, Anderson Rodrigues Lima Caires, Rozanna Marques Muzzi

**Affiliations:** ^1^Faculty of Exact Sciences and Technology, Federal University of Grande Dourados, Dourados, MS, Brazil; ^2^Faculty of Medical Sciences, State University of Campinas, Campinas, SP, Brazil; ^3^Institute of Chemistry, State University of Campinas, Campinas, SP, Brazil; ^4^Institute of Physics, Federal University of Mato Grosso do Sul, Campo Grande, MS, Brazil

## Abstract

The techniques used to monitor the quality of the biodiesel are intensely discussed in the literature, partly because of the different oil sources and their intrinsic physicochemical characteristics. This study aimed to monitor the thermal degradation of the fatty acid methyl esters of *Sesamum indicum* L. and *Raphanus sativus* L. biodiesels (SILB and RSLB, resp.). The results showed that both biodiesels present a high content of unsaturated fatty acids, ∼84% (SILB) and ∼90% (RSLB). The SILB had a high content of polyunsaturated linoleic fatty acid (18  :  2), about 49%, and the oleic monounsaturated (18  :  1), ∼34%. On the other hand, RSLB presented a considerable content of linolenic fatty acid (18  :  3), ∼11%. The biodiesel samples were thermal degraded at 110°C for 48 hours, and acid value, UV absorption, and fluorescence spectroscopy analysis were carried out. The results revealed that both absorption and fluorescence presented a correlation with acid value as a function of degradation time by monitoring absorptions at 232 and 270 nm as well as the emission at 424 nm. Although the obtained correlation is not completely linear, a direct correlation was observed in both cases, revealing that both properties can be potentially used for monitoring the biodiesel degradation.

## 1. Introduction

The human interest in using the biomass available on the planet has been increased due to the global population growth, accelerated industrial development, reduced global petroleum reserves, and concerns about environmental impacts [1]. In this context, the search for alternative sources of renewable energy has growing interest. Besides renewable, the alternative energy sources should also be ecologically correct, socially sustainable, and economically viable. Clearly, biofuels compose part of the alternative ecologically friendly energy because they are produced from renewable sources and also more friendly to the environment than fossil-derived fuels [2].

Typically, oleaginous species are used as the main source of raw materials to produce biofuel [3]. Biodiesels can be produced by different oleaginous sources depending on the raw material available in each country and/or region. Consequently, it have been reported that biodiesels can be synthesized from soybean, corn, canola, palm, animal fats, recycled greases, and others [4, 5]. For instance, in 2016/2017 soybean oil was the main feedstock for biodiesel production worldwide (341 million tons), for a total of 558 million tons of total oilseeds [6].

The desirable characteristics of the raw materials for biodiesel production include (i) adaptability to local growing conditions; (ii) regional availability; (iii) high oil content; (iv) favorable fatty acid composition; (v) compatibility with existing agricultural practices; (vi) agricultural inputs; (vii) by-product markets; (viii) land compatibility; and (ix) rotational adaptability with other cultures [7].

Nevertheless, depending on the region, different vegetable oils are available to biodiesel production according to the agricultural, social, and commercial factors as previously mentioned. As consequence, different biodiesels are produced with particular chemical compositions, such as secondary compounds (polyphenols, carotenoids, and chlorophylls) and triacylglycerols (TAGs) with different lengths and unsaturation contents of the fatty acid carbon chain. The secondary compounds and TAG's compositions are directly associated with the susceptibility of biodiesel degradation: (i) secondary compounds (antioxidants) can promote biodiesel stability, and (ii) unsaturated carbon chain content makes biodiesel more susceptible to the oxidation [8].

The biodiesel commercialization is dependent of several quality parameters [9]. Among them, acid value (AV) is an important parameter to evaluate the biodiesel characteristics. This parameter indicates the formation of organic acids from TAG's oxidation by molecular oxygen [10]. High AV indicates that biodiesel is very degraded, and its commercialization is impaired [11].

In the course of biodiesel degradation process, several small molecules are generated, such as peroxides, aldehydes, ketones, and carboxylic acids, among others [9]. These molecules are formed by reaction between carbon chains of biodiesel with singlet oxygen, forming oxidized compounds [12]. Recent studies have demonstrated the potential of the spectroscopic technique in determining some important transformations in the molecular structure of the alkyl esters from biodiesel during degradation process [13, 14]. Despite the fact that the formed degradation, specifically carboxylic acids, be satisfactory quantified by the titration method (AV parameter), it remains unclear the relation of generating these compounds with biodiesel light absorption/emission.

Our previous works have shown that the acidification of vegetable oils reflects on the changes in the absorbances at 232 and 270 nm with direct impact on the emission profile [15, 16]. However, the optical behavior is still not completely understood for biodiesel, especially for unconventional vegetable sources studied here (from *Raphanus sativus* L. and *Sesamum indicum* L. seeds). In this sense, the present study aimed to apply fluorescence spectroscopy for monitoring biodiesel degradation and determine its correlation with acid value and UV absorbance changes.

## 2. Materials and Methods

### 2.1. Chemicals, Materials, and Equipment

Methanol (Impex; 99.8%); KOH (Dinâmica; 95%); MgSO_4_ (Impex; 98%); ethyl ether (Impex; 98%); ethyl alcohol (Dinâmica; 96%); hexane (Panreac; 95%); laboratory oven (Sterilifer SXCR42; 42 liters); hydraulic press (Ecirtec-mpe40); gas chromatograph (GC-2012-plus-Shimadzu); 743-Rancimat Metrohm; UV-Vis spectrometer (Cary 50 Varian); and spectrofluorimeter (Cary Eclipse Varian) were used.

### 2.2. Oil Extraction

*Raphanus sativus* L. (RSL) and *Sesamum indicum* L. (SIL) seeds were commercially obtained from local companies. The seeds were dehydrated in laboratory oven under temperature of 60°C for 14 hours. Then, the seeds were submitted to hydraulic press extraction obtaining a yield of extracted oil of 32% (w/w) and 45% (w/w) for RSL and SIL oils, respectively. The extracted oils were kept in a dark environment (refrigerator) at −4°C until methyl esters synthesis. Unrefined crude oils were used in all analyses.

### 2.3. Biodiesel Production

Primary, the acid values for RSL and SIL oils were titrated, obtaining the values of 0.35 and 0.82 mg KOH/g, respectively. The biodiesel was obtained by the transesterification of the extracted crude oils, using the potassium methoxide as a catalyst. Initially, the potassium methoxide was obtained by reacting 40 mL of methanol with 1.8 g of KOH under stirring at 60°C for 30 min. After that 90 g of oil was added in the solution and remained under stirring at 60°C during approximately 2 h for the transesterification reaction. This reaction was monitored by thin layer chromatography as described in our recent study [17, 18]. After the transesterification process, the mixture was decanted to separate the biodiesel from the by-product glycerol. The phase containing the esters of interest was washed with distilled water and then dried with anhydrous MgSO_4_, filtered and concentrated in a rotary evaporator at 55°C for approximately 3 h [15].

### 2.4. Sample Degradation

The degradation was carried out as follows: (i) the biodiesel samples were weighed in amber flasks, approximately 3.5 g of sample (in each flask) in which the samples were prepared in triplicate; (ii) all samples were subjected to heating in an air circulation laboratory oven at 110°C for 48 hours; (iii) initially, flasks were removed and stored (*n* = 3) every 2 hours up to the 24 hour period, and finally, flasks were sampled in 36 and 48 hours. The samples were stored at approximately −4°C until analyses. The biodiesel oxidation was governed by the biodiesel-atmospheric air interaction without any additional air flow rate. The biodiesel-air contact was defined simply by the size of the flask aperture, a radius of approximately 0.8 cm with 2.0 cm^2^ of area with constant air renovation. All analyses were performed in triplicates considering three independent flasks containing degraded biodiesel samples.

### 2.5. Fatty Acid Composition

The analyses of the fatty acid composition were performed by using a gas chromatography system according to the method Ce 2-66 [19]. The fused silica SP-2560 column (100 m and 0.25 mm) was used in the separation process. The isothermal temperature was programmed at 140°C for 5 min followed by heating at 4°C min^−1^ up to 240°C, remaining at this level for 30 min. The temperature of the vaporizer was 250°C and detector was 260°C, using helium as carrier gas.

### 2.6. Oxidative Stability Study

The oxidative stability, expressed by the induction period (IP) in hours, was determined in duplicate by a Rancimat apparatus according to the EN 14112 method. Samples weighing 3.0 g (±0.1) were added into a sealed glass vessel reaction, heated at constant temperature of 110°C, and analyzed under a constant air flow rate (10 L·h^−1^) passing through the samples and then into measuring vessel containing 50 mL ultrapure water [20].

### 2.7. Acid Value Analysis

The variation of total acid value during the degradation period was evaluated by using the classical titrations. In three Erlenmeyers were added 2.0 g of sample, 25 mL of the solution ethyl ether  :  ethyl alcohol (2  :  1), and two drops of the phenolphthalein indicator. The samples were then titrated with 0.1 mol·L^−1^ potassium hydroxide, duly standardized, until the appearance of the pink coloration observed for at least 30 seconds [21].

### 2.8. UV Absorption and Fluorescence Spectroscopy Analysis

All samples were diluted in a hexane HPLC grade. The concentration was 0.025% (w/v) and 0.030% (w/v) for the biodiesels produced from *S. indicum* L. (SILB) and *R. sativus* L. (RSLB), respectively. Molecular absorption and emission analyses were performed at room temperature using a 10 mm thick quartz cell.

### 2.9. Statistical Analyses

The statistical analyses were performed by two-way ANOVA followed by the Tukey's (*P* < 0.05) test using GraphPad Prism (GraphPad 164 Software, Version 6.0, San Diego, CA).

## 3. Results and Discussion

### 3.1. Fatty Acid Composition

Table 1 shows the composition of fatty acids present in each oilseed. The results show that both feedstocks have a high content of unsaturated fatty acids, ∼84% for *S. indicum* L. and ∼90% for *R. sativus* L. As can be clearly seen, SIL has a higher content of linoleic polyunsaturated fatty acid (18  :  2) and oleic monounsaturated fatty acid (18  :  1), which were approximately 49% and 34%, respectively. On the other hand, RSL presented a considerable content of linolenic fatty acid (18  :  3), ∼11%. It was also observed that RSL presented two monounsaturated fatty acids, which were not found in SIL (eicosenoic acid (20  :  1) and erucic acid (22  :  1)). Moreover, significant quantities of oleic (18  :  1), >32%, for both feedstocks were quantified. The fatty acid composition in the feedstock is very important because the biodiesel degradation can be estimated by knowing the type and quantity of fatty acids. This estimation is possible considering the unsaturations present in the carbon chains from fatty acids because these regions are susceptible to atmospheric oxygen attack. The oxygen reaction leads to the breaking of these chains to form unwanted oxidized compounds that change the initial physicochemical properties of the samples [22]. However, similar quantities of unsaturated fatty acids (∼84% for SIL and ∼90% for RSL) may not reflect an identical profile of degradation due to the presence of secondary compounds (intrinsic antioxidants), depending on its chemical feature and content in the oilseeds [23, 24].

### 3.2. Acid Value Determination

**Figure 1:**
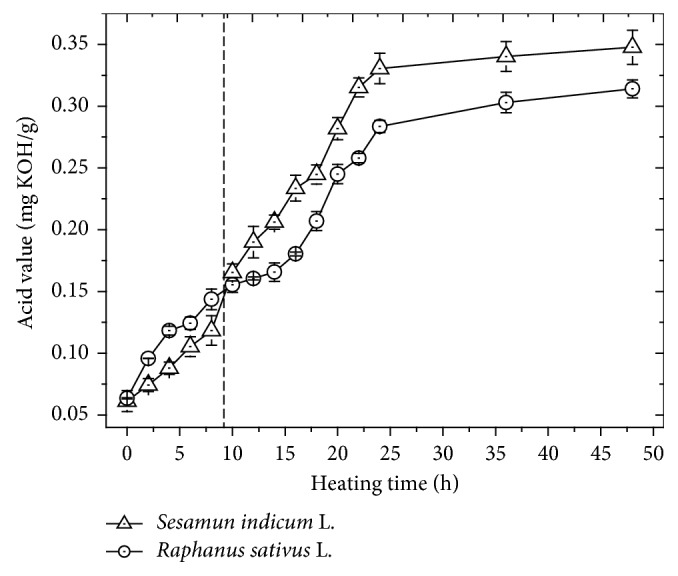
Acid values for biodiesels from *S. indicum* L. and *R. sativus* L. during the thermal degradation process. Error bars represent standard deviations.

The monitoring of the degradation process was carried out by the quantification of the acidic compounds (Figure 1). In general, a similar profile for the formation of acidic compounds in both biodiesels was observed. There is an increase in the formation of these compounds in the first 24 hours of thermodegradation, tending to a plateau with a AV of ∼0.35 and ∼0.32 mg KOH/g for SILB and RSLB, respectively, until 48 hours. It is interesting to note that there is a change in the acidity profile between these two biodiesels in approximately 9 hours, presenting a relative inversion. This means that the RSLB presents lower susceptibility to oxidation and formation of acidic compounds than SILB after 9 h of induced degradation. The difference in the acid values from SILB to RSLB may be related to the major content of linoleic acid (18  :  2), approximately 49% present in the SIL fatty acid composition. This trend makes the biodiesel derived from SIL more susceptible to degrade than biodiesel from RSL. In this process, the small organic acids can be formed from radical reactions; in which, the oxygen reacts with the unsaturations forming organic hydroperoxides, further the formation of aldehydes and finally oxidized to a stable product such as carboxylic acid [22, 25]. In summary, Figure 1 shows that although the degradation profiles are not equal, a similar response of acid value analyses was observed for both SILB and RSLB.

### 3.3. UV Absorption Measurement

The carbonic chains of the methyl esters undergo oxidation exhibit absorption of light at two characteristic wavelengths, at 232 nm and 270 nm. These wavelengths refer to modifications in the structures of the carbon chains and/or the formation of new molecules (degradation products). At the initial stage of degradation, there is a more prominent increase in absorption at 232 nm (primary oxidation), which occurs due to the formation of conjugated dienes. At the end of the degradation process, there is a more prominent increase in absorption at 270 nm (secondary oxidation), associated with aldehydes, ketones, and carboxylates [26, 27].

**Figure 2:**
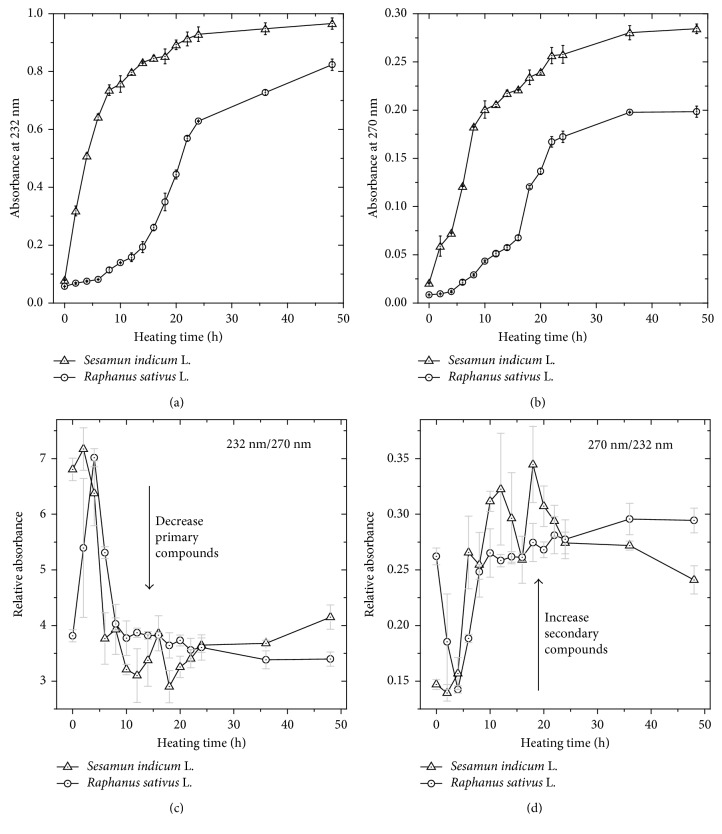
Absorbance values at (a) 232 nm and (b) 270 nm for biodiesels from *S. indicum* L. and *R. sativus* L. during the thermal degradation process. (c) and (d) show the absorbance ratios with formation of secondary compounds after ∼10 h of thermal degradation. Error bars represent standard deviations.

In Figure 2(a), it is possible to see the formation of the primary compounds in the biodiesels. There was a higher formation of these compounds in SILB in the first 10 hours of induced degradation. On the other hand, RSLB presents lower production of these primary compounds in the first 15 hours, being formed more intensely after 24 hours. It is interesting to observe a similar trend for the response of the measured absorbance at 270 nm (Figure 2(b)), comparing with the absorbance at 232 nm. This means that the primary and secondary compounds are generated proportionally in both biodiesels [28]. Moreover, the higher absorption at 232 and 270 nm for SILB can be attributed to its composition in terms of linoleic acids.

Previous studies have reported that an estimate of the level of the biodiesel oxidation can be obtained by analyzing the absorbance ratio of 232 to 270 nm [26, 27, 29]. Figures 2(c) and 2(d) show that both biodiesels form large amounts of more oxidized compounds after ∼10 hours during the thermal degradation. This response can be understood by the similarity in the unsaturated fatty acids for both biodiesels (∼84% for SIL and ∼90% for RSL).

**Figure 3:**
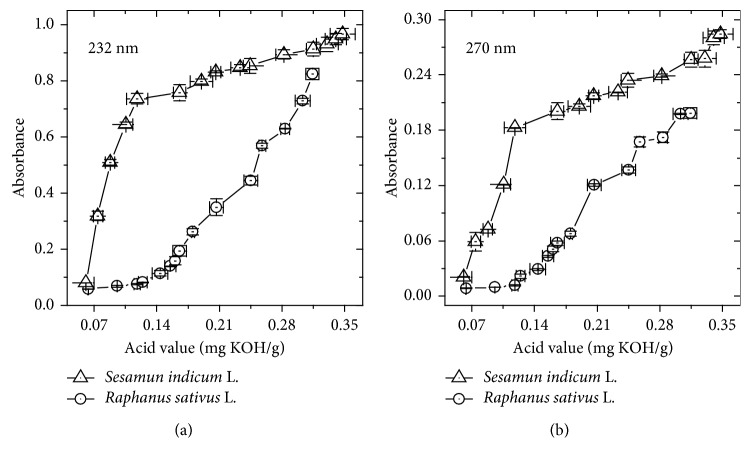
Correlation between the acid values and the absorbance values at (a) 232 nm and (b) 270 nm for the biodiesels from *S. indicum* L. and *R. sativus* L. Error bars represent standard deviations.

In addition, the correlation behavior between the quantified acid values and the absorbance responses of the primary and secondary compounds generated in the oxidative process was performed.  Figure 3 shows that, in general, considering at both wavelengths, there is a direct, but not linear correlation in the values for both biodiesels. Moreover, an opposite behavior was observed for each biodiesel. The inclination of the correlation curve is greater for SILB than for RSLB, suggesting a higher sensitivity of absorbance measurements comparatively to acid value titration for the SILB. In this sense, the results suggest that the oxidative stage of the SILB can be better studied by monitoring the alterations in the UV absorbances. Differently, RSLB degradation can be evaluated with similar sensitivity applying AV analysis or UV absorption at 232 or 270 nm.

### 3.4. Fluorescence Profiles of Biodiesels

**Figure 4:**
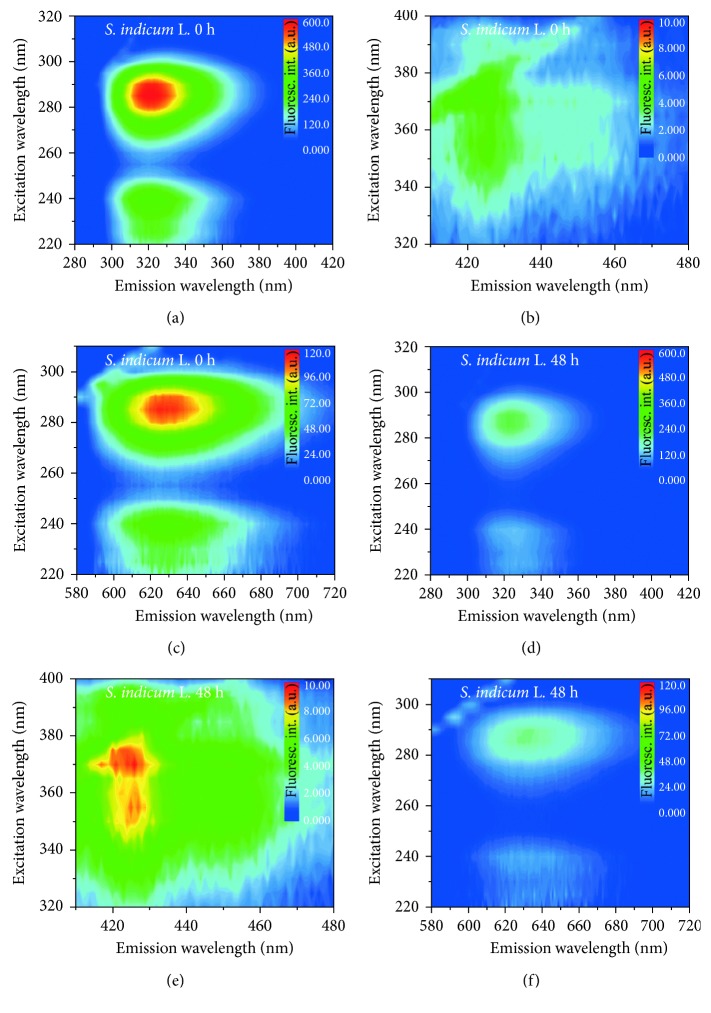
Fluorescence intensity maps of *S. indicum* L. biodiesel (a, b, c) before the oxidative process and (d, e, f) after 48 h of degradation. Scattering not omitted.

**Figure 5:**
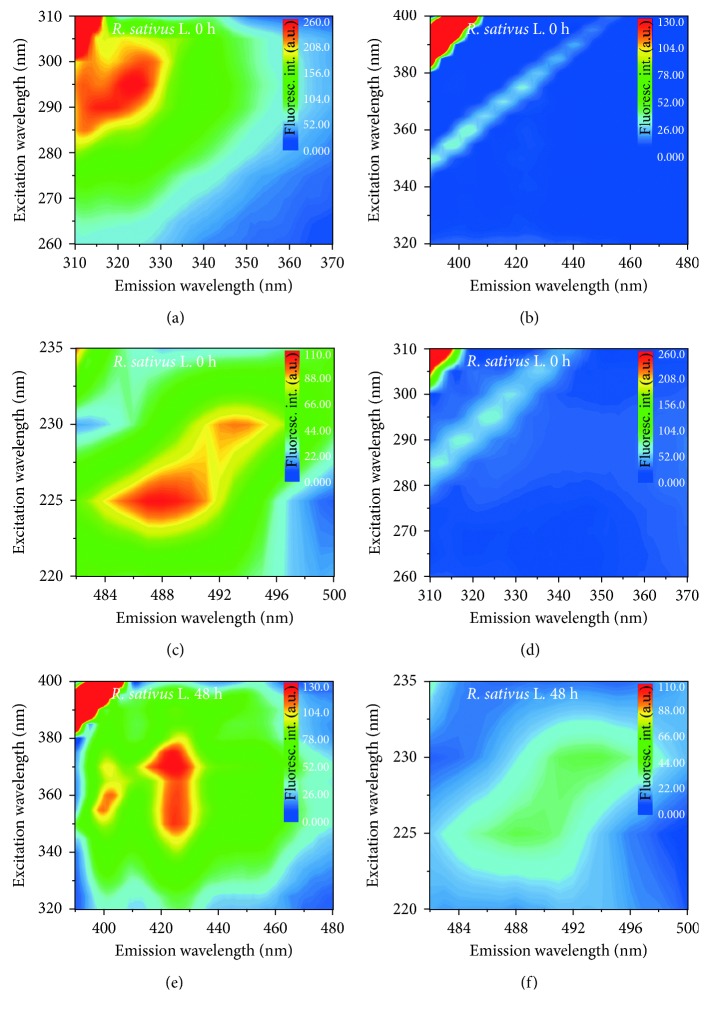
Fluorescence intensity maps of *R. sativus* L. biodiesel before the oxidative process and after 48 h of degradation. Scattering not omitted.

Alternatively, the excitation/emission profile was investigated through the contour maps presented in Figures 4 and 5 for SILB and RSLB, respectively.  Figure 4 shows changes in fluorescence intensities from 280 to 720 nm with excitation of 220 to 400 nm. Figures 4(a) and 4(d) reveal that the oxidation process induced a strong reduction in the emission intensity at ∼320 nm (excitations at ∼235 and ∼285 nm). Similarly, a decrease in the emission intensity at ∼630 nm, when excited at 235 and 285 nm, was also observed as presented in Figures 4(c) and 4(f). This last emission is attributed to chlorophylls [27, 30], clearly degraded under thermal conditions. Additionally, Figures 4(b) and 4(e) show that an increase in the emission intensity in the 415–430 nm range was determined, with excitation from 345 to 380 nm, revealing the production of fluorescent compounds during the degradation process. This behavior is similar to that observed by other studies and corroborates with our previous studies which demonstrated that conjugated tetraenes are produced during the biodiesel degradation [31, 32].

Figure 5 shows changes in fluorescence intensities from 310 to 500 nm, with excitation of 220 to 400 nm for RSLB. Figures 5(a) and 5(d) show the reduction in the emission intensities at ∼320 nm. According to the literature, the emission at 322 nm region is related to the natural antioxidant tocopherols [33]. The results also revealed a decrease in the emission intensity from ∼480 to ∼500 nm as presented in Figures 5(c) and 5(f). This emission region was recently reported to be associated with the carotenoids as reported by Silva et al. [30]. Similarly to that observed for SILB, it was possible to observe the appearance of emissions at ∼425 nm, directly associated with the degradation compounds. As previously discussed, this emission is associated with the conjugated tetraenes which are formed during the degradation of the methyl esters [32, 34].

**Figure 6:**
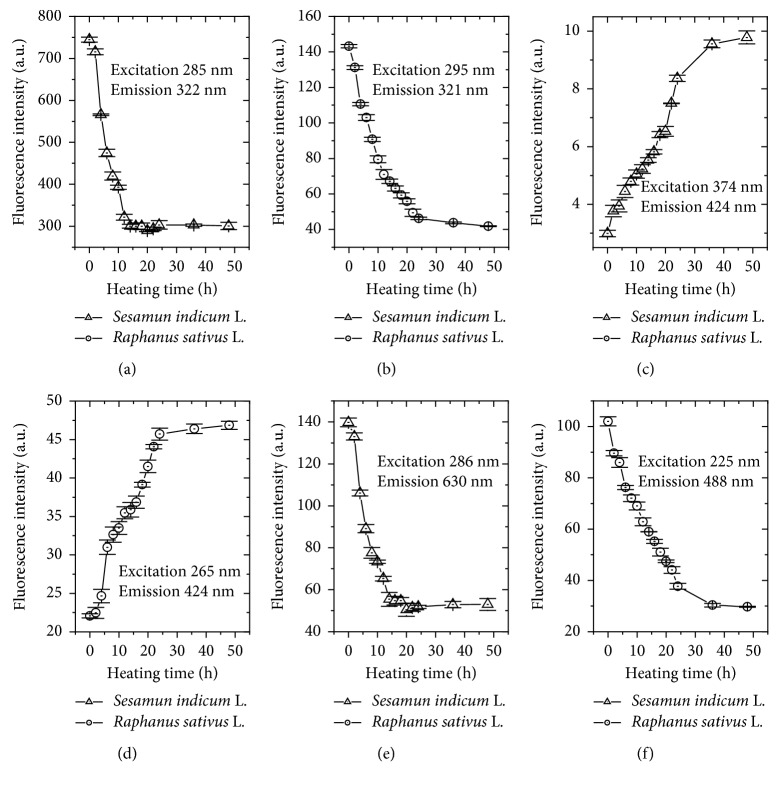
Fluorescence intensities of biodiesels from *S. indicum* L. and *R. sativus* L. during the thermal degradation process at specific wavelengths: (a) emission at ∼322 and (b) emission at 321 nm; (c, d) emission at 424 nm; (e) emission at 630 nm; (f) emission at 488 nm. Error bars represent standard deviations.

From the emission/excitation contour maps, some candidate wavelengths were selected to monitor the oxidative process of SILB and RSLB, as presented in Figure 6. The fluorescence profile of SILB shows that the emission intensity at 322 nm decays rapidly to ∼300 a.u. in the first 10 hours (Figure 6(a)). For RSLB, it was observed that the intensity decrease is less abrupt (Figure 6(b)), but equally significant. In addition, it is interesting to note the convergence of fluorescence intensity to specific values. Moreover, these behaviors are very similar to that observed for AV analysis and UV absorbances profiles, with change on the response at approximately 10 hours of degradation time. Similar decrease of emission may be observed at wavelengths close to 630 nm for SILB limiting to the initial 15 hours of the degradation process (Figure 6(e)). For RSLB, the decrease in emission at 488 nm occurs similarly to that observed for emission at 321 nm with satisfactory response in the initial 24 hours of the oxidative process (Figure 6(f)). On the other hand, the oxidation process may be monitored by increase of the emission at 424 nm, being a very promising parameter to monitor the oxidative process of both biodiesels (Figures 6(c) and 6(d)).

### 3.5. Fluorescence, Absorbance, and Acid Value Correlations

**Figure 7:**
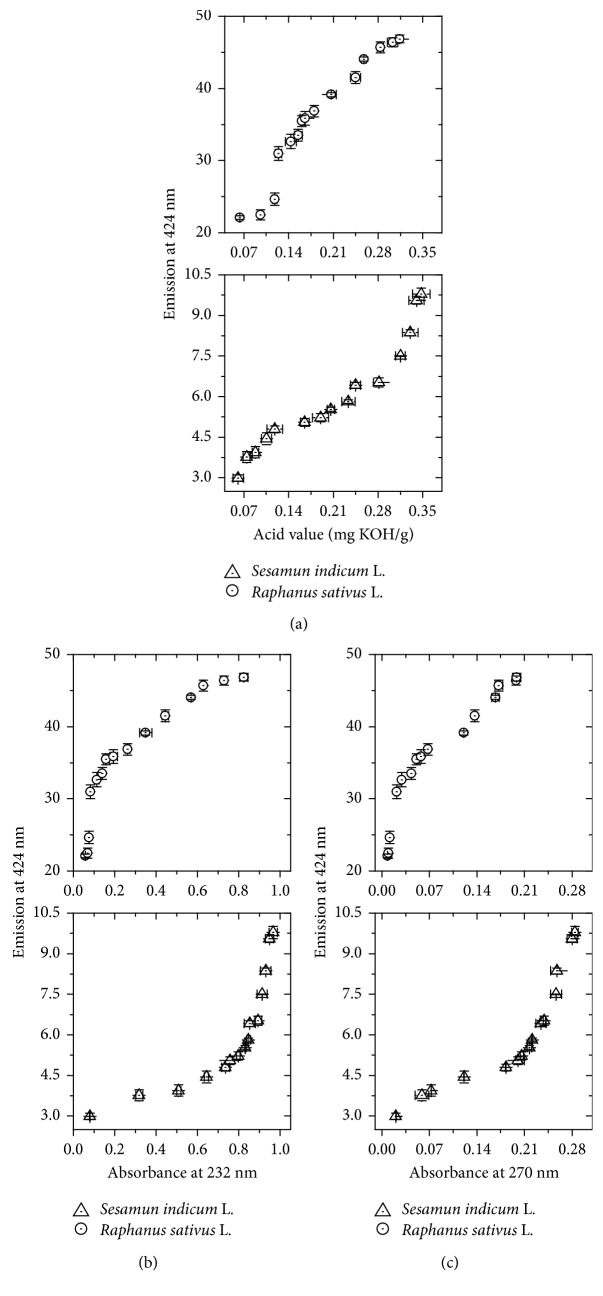
Correlation between emission at 424 nm (excitation at 374 and 265 nm for *S. indicum* L. and *R. sativus* L., resp.) and (a) acid value, (b) absorbance at 232 nm, and (c) absorbance at 270 nm for SILB and RSLB, respectively. Error bars represent standard deviations.

The emission profile at 424 nm may be associated with classical technique acid value and more recently with absorptions at 232 and 270 nm [16].  Figure 7(a) shows the correlation of fluorescence intensity at 424 nm with acid values for RSLB and SILB, respectively. These results show that there is a direct correlation in both cases, indicating that the emission at 424 nm is a potential tool for monitoring the oxidative stability for both SILB and RSLB. These correlations will be better addressed in Section 3.6.

Figures 7(b) and 7(c) show the obtained correlations of emission at 424 nm with absorbance at 232 and 270 nm, respectively. The results present a direct but not linear correlation. Furthermore, interesting to note different correlation plotted for each biodiesel. These results can be associated with specific functions, such previously demonstrated [16]; however, it is clear that there is a nonlinear correlation.

### 3.6. AV Titration versus Emission at 424 nm

**Figure 8:**
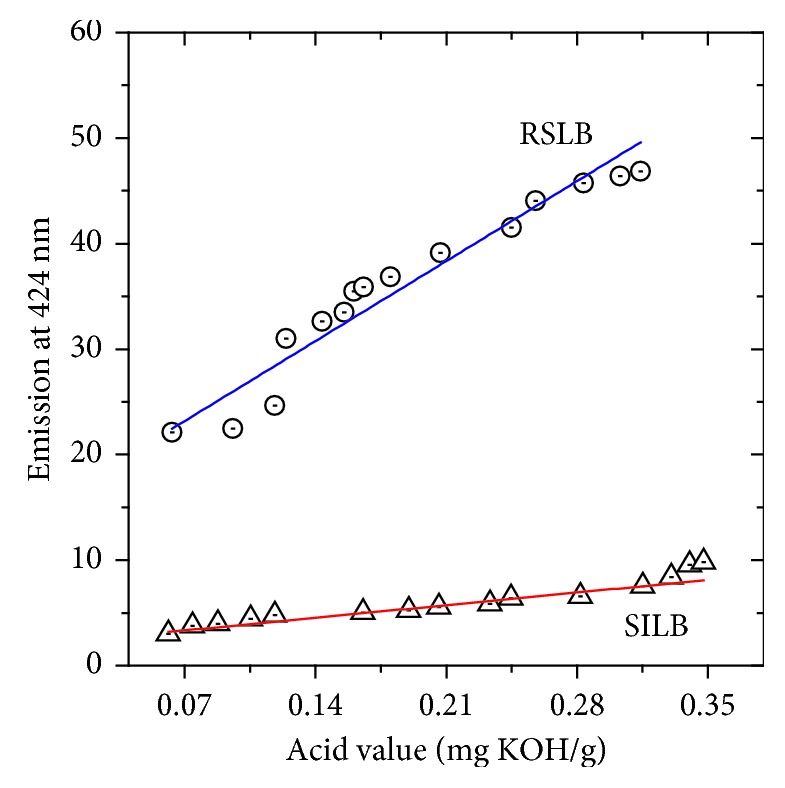
Fitting for the linear correlation between emission at 424 nm and acid value for RSLB and SILB.

To evaluate the potential of molecular fluorescence spectroscopy for monitoring the degree of oxidation of the biodiesel samples, a close analysis of the correlation between fluorescence intensity at 424 nm and acid value was performed as shown in Figure 8. Clearly, fluorescence emission at 424 nm can be used to estimate the acid values using linear equations (1) and (2), respectively, which were obtained from the data fitting presented in Figure 8:

(1) $$\begin{array}{c} {\mathrm{Em}}_{424}=15.6+108.3*\mathrm{AV}, \end{array}$$

(2) $$\begin{array}{c} {\mathrm{Em}}_{424}=2.1+17.1*\mathrm{AV}. \end{array}$$

### 3.7. Consideration about Oxidative Stability of Biodiesels

The induction periods determined in this work for biodiesels from SILB and RSLB were (0.07 ± 0.01) h and (1.60 ± 0.10) h, respectively. These results show that RSLB is more stable than SILB. In fact, in Figure 2, it was possible to see that the formation of primary and secondary compounds of degradation is more rapidly formed for SILB than RSLB; a possible explanation may be centered on the higher content of polyunsaturated fatty acids present in the SILB, making SILB more susceptible to degradation.

The classical method for evaluating the stability of the biodiesels is the Rancimat method [35]. However, several alternatives methods were proposed to increase the knowledge about a particular sample, adding with information obtained from more classical analyses such as titration of peroxide, iodine, and acid values [36]. Other methods, such as infrared spectroscopy, PetroOXY, ultrasonic-accelerated oxidation, differential scanning calorimetry (DSC), and thermogravimetric analysis (TGA) [8, 37], have been also recently applied to monitor the biodiesel quality. Each of these techniques can provide specific sample diagnostic which they should be crossed and interpreted to obtain a detailed picture of the biodiesel quality condition. In this context, the present study shows that due to the close correlation between biodiesel emission and acid values, fluorescence spectroscopy can be potentially applied to monitoring the biodiesel degradation.

## 4. Conclusion

This work showed that the composition of fatty acids was feedstock dependent, with high content of unsaturated fatty acids, principally polyunsaturated in SILB. The high content of unsaturated fatty acids reflects in the transformations undergone by RSLB and SILB carbon chains accused by acid value analysis, UV absorption, and fluorescence analysis.

The oxidation stages of the biodiesels were monitored by the formation of acidic compounds through the classic determination of the acid value, obtaining a similar change for both biodiesels. It was also found that the absorption profiles at 232 and 270 nm were more sensible to discriminate the biodiesels from different feedstocks under degradation process, evidenced by the correlation between the absorbance and acid value analysis. Alternatively, our results showed that molecular fluorescence spectroscopy can be used for monitoring the degradation stages of the RSLB and SILB according to the linear correlation between the emission at 424 nm and AV. Finally, it was demonstrated that the acid value for both biodiesels can be predicted by analyzing the emission at 424 nm. We summarize that the present findings revealed that fluorescence spectroscopy can be potentially used to monitor the biodiesel degradation.

## Figures and Tables

**Table 1:** Fatty acid methyl ester composition in biodiesels from *Sesamum indicum* L. and *Raphanus sativus* L. [16].

Fatty acids (%)	Description	*S. indicum* L.	*R. sativus* L.
Palmitic	C 16 : 0	8.54 ± 0.03	6.38 ± 0.66
Palmitoleic	C 16 : 1	0.00 ± 0.00^A^	0.33 ± 0.01^A^
Stearic	C 18 : 0	6.83 ± 0.01	2.32 ± 0.14
Oleic	C 18 : 1(n-9)	34.12 ± 0.09	31.72 ± 2.14
Vaccenic	C 18 : 1(n-7)	0.00 ± 0.00^B^	0.00 ± 0.00^B^
Linoleic	C 18 : 2(n-6)	48.86 ± 0.08	18.35 ± 1.27
Arachidic	C 20 : 0	0.87 ± 0.03^C^	0.93 ± 0.06^C^
Eicosenoic	C 20 : 1	0.18 ± 0.01	8.80 ± 0.59
Linolenic	C 18 : 3(n-3)	0.41 ± 0.00	11.38 ± 0.67
Docosanoic	C 22 : 0	0.19 ± 0.01^D^	0.53 ± 0.03^D^
Erucic	C 22 : 1	0.00 ± 0.00	17.89 ± 1.13
Lignoceric	C 24 : 0	0.00 ± 0.00^E^	0.63 ± 0.04^E^
Nervonic	C 24 : 1	0.00 ± 0.00^F^	1.80 ± 0.29^F^

Σ saturated	—	**16.43 ± 0.02**	**10.79 ± 0.93**
Σ monounsaturated	—	**34.30 ± 0.09**	**60.54 ± 4.16**
Σ polyunsaturated	—	**49.27 ± 0.08**	**29.73 ± 1.94**

Values are mean ± sd (standard deviation) (*n* = 3) expressed as a percentage in relation to total fatty acids quantified. Values on the same line highlighted by equal superscript letters indicate no significant differences (*P* < 0.05).
